# Complete Genome Sequences of 17 Rapidly Growing Nontuberculous Mycobacterial Strains

**DOI:** 10.1128/genomeA.01009-16

**Published:** 2016-09-22

**Authors:** Lindsay J. Caverly, Theodore Spilker, John J. LiPuma

**Affiliations:** Department of Pediatrics, University of Michigan Medical School, Ann Arbor, Michigan, USA

## Abstract

We report the complete genome sequences of 17 rapidly growing nontuberculous mycobacterial (NTM) strains, including 16 *Mycobacterium abscessus* complex strains and one *M. immunogenum* strain. These sequences add value to studies of the genetic diversity of rapidly growing NTM strains recovered from human specimens.

## GENOME ANNOUNCEMENT

The *Mycobacterium abscessus* complex (*M. abscessus* subsp. *abscessus*, *M. abscessus* subsp. *massiliense*, and *M. abscessus* subsp. *bolletii*) and *M. immunogenum* are closely related, rapidly growing nontuberculous mycobacteria (NTM) that cause skin, soft tissue, and pulmonary infections (1, 2). Comparative genomic analyses have provided valuable information about the epidemiology of rapidly growing NTM infections, including identification of global outbreak strains of the *M. abscessus* complex (3) and potential patient-to-patient transmission of *M. abscessus* complex strains among individuals with cystic fibrosis (4). Here, we report the complete genome sequences of six *M. abscessus* subsp. *abscessus* strains, ten *M. abscessus* subsp. *massiliense* strains, and one *M. immunogenum* strain, all isolated from human sources. These complete genome sequences will add value to studies of genetic diversity within clinically isolated, rapidly growing NTM.

Bacteria were grown on Middlebrook 7H11 agar at 37°C. The bacterial culture was resuspended in 1 ml of ultrapure water and adjusted to an optical density of 0.45, corresponding to approximately 10^9^ CFU. Genomic DNA was extracted from 400 µL of the adjusted suspension using the MagNA Pure Compact nucleic acid isolation kit (Roche, Indianapolis, IN, USA) following the manufacturer’s instructions. Genomic DNA libraries were prepared using an Illumina TruSEQ DNA library kit and sequenced on an Illumina HiSEQ 4000 paired-end flow cell (2 × 150-bp read length, V4 chemistry) at the University of Michigan Medical School DNA Sequencing Core. Output files containing FASTQ reads were checked and edited using Trimmomatic version 0.33 (5). Read-correction and assembly of the draft genomes were carried out using SPAdes version 3.5.0 (6). The draft genomes were aligned with the 13 completed and annotated *M. abscessus* complex and *M. immunogenum* genomes currently available in NCBI (http://www.ncbi.nlm.nih.gov/genome/browse) using the Parsnp and Gingr programs of the Harvest tools version 1.2 suite (7).

For each strain, the contigs were ordered using the closest genetically similar strain as the reference genome, either *M. abscessus* ATCC 19977^T^ (8), *M. massiliense* strain CCUG 48898^T^ (9, 10), or *M. immunogenum* CCUG 47286^T^ (11), with Mauve version 2.4.0 (12). The sorted draft genomes were then manually gap-filled by identifying short segments (15 to 25 bp) on the ends of the two contiguous pieces that matched both ends of a single contig within the draft genome not already included by Mauve in the alignment. These matches were verified by obtaining the longest possible perfect match on both sets of ends, checked with BLASTn for continuity, confirmed with BLASTx when possible, and checked for the appropriateness of gap distance against the nearest complete reference strain genome. The genomes were annotated using NCBI’s whole-genome shotgun submission portal containing the automated Prokaryotic Genomic Annotation Pipeline (PGAP) option. The completed genomes ranged in size from 4,799,801 bp to 5,604,845 bp, and the number of conserved domain sequences ranged from 4,629 to 5,437 (Table 1).

**TABLE 1:** Global statistics of complete genome sequences of *Mycobacterium abscessus* complex and *M. immunogenum* strains^*a*^

Isolate	Identification	Year of isolation	BioSample no.	Accession no.	Genome size (bp)	CDSs^*b*^ (total)
FLAC004	*M. abscessus* subsp. *massiliense*	2012	SAMN04572934	CP014951	5,242,371	5,191
FLAC005	*M. abscessus* subsp. *massiliense*	2012	SAMN04572935	CP014952	4,869,298	4,696
FLAC006	*M. abscessus* subsp. *massiliense*	2012	SAMN05244437	CP016188	4,891,993	4,731
FLAC007	*M. abscessus* subsp. *massiliense*	2013	SAMN04572936	CP014953	5,064,478	4,967
FLAC008	*M. abscessus* subsp. *massiliense*	2013	SAMN04572937	CP014954	5,166,100	5,085
FLAC013	*M. abscessus* subsp. *abscessus*	2013	SAMN04572938	CP014955	5,074,222	4,920
FLAC016	*M. immunogenum*	2013	SAMN05244438	CP016189	5,604,845	5,437
FLAC028	*M. abscessus* subsp. *abscessus*	2013	SAMN05244439	CP016190	5,188,101	5,067
FLAC029	*M. abscessus* subsp. *abscessus*	2013	SAMN04572939	CP014956	5,188,507	5,063
FLAC030	*M. abscessus* subsp. *massiliense*	2013	SAMN05244440	CP016191	4,867,257	4,754
FLAC031	*M. abscessus* subsp. *abscessus*	2013	SAMN04572940	CP014957	5,146,255	4,990
FLAC045	*M. abscessus* subsp. *massiliense*	2015	SAMN04572941	CP014958	5,217,908	5,170
FLAC046	*M. abscessus* subsp. *abscessus*	2015	SAMN05244441	CP016192	5,214,168	5,094
FLAC048	*M. abscessus* subsp. *massiliense*	2015	SAMN04572942	CP014959	4,939,234	4,788
FLAC049	*M. abscessus* subsp. *abscessus*	2015	SAMN04572943	CP014960	4,799,801	4,629
FLAC054	*M. abscessus* subsp. *massiliense*	2015	SAMN04572944	CP014961	5,330,954	5,242
FLAC055	*M. abscessus* subsp. *massiliense*	2015	SAMN05244442	CP016193	5,331,134	5,241

a All strains are included in BioProject PRJNA315990 and SRA SRP072743.

b CDSs, coding sequences.

### Accession number(s).

This whole-genome shotgun project has been deposited in GenBank under the accession numbers listed in Table 1. The versions described in this paper are the first versions.
